# Are comparisons of mental disorders between Chinese and German students possible? An examination of measurement invariance for the PHQ-15, PHQ-9 and GAD-7

**DOI:** 10.1186/s12888-020-02859-8

**Published:** 2020-10-01

**Authors:** Yan Zhou, Jing Xu, Winfried Rief

**Affiliations:** 1 Clinical Psychology and Psychotherapy, Department of Psychology, Philipps-University of Marburg, Gutenbergstr. 18, D-35032 Marburg, Germany; 2 Department of Marxism, University of Zhengzhou, Zhengzhou, China

**Keywords:** Cross-cultural comparison, Measurement invariance, Patient Health Questionnaire-15 (PHQ-15), Patient Health Questionnaire-9 (PHQ-9), Generalized Anxiety Disorder-7 (GAD-7)

## Abstract

**Background:**

The Patient Health Questionnaire (PHQ) is one of the most commonly used instruments to assess mental disorders. However, research on its cross-cultural measurement invariance is not yet sufficient. This study examined the measurement invariance of the Chinese and German versions of the PHQ’s somatic symptom severity scale (PHQ-15), depressive symptom severity scale (PHQ-9) and seven-item Generalized Anxiety Disorder (GAD-7) scale as a prerequisite for their use in cross-cultural comparisons.

**Methods:**

We used online data collected from groups of Chinese students in China (*n* = 413) and German students in Germany (*n* = 416). Separate measurement models for each group were examined using confirmatory factor analysis and measurement invariance testing was conducted to test the cross-cultural equivalence.

**Results:**

Findings demonstrated that the PHQ-9 and GAD-7 had partial scalar measurement invariance, but the cross-cultural measurement invariance of the PHQ-15 could not be confirmed. Comparisons of latent means did not indicate differences in the levels of depression and anxiety symptoms between Chinese and German samples.

**Conclusion:**

The PHQ-9 and GAD-7 can be used in cross-cultural comparison of prevalence, but the intercultural use of PHQ-15 is more problematic. Findings are discussed from intercultural and methodological perspectives.

## Background

Depression, anxiety disorders and somatoform disorders are the most common mental disorders worldwide and differences in epidemiology exist across countries and cultures [1, 2]. Previous studies showed that base rates of depression and anxiety disorders are lower in China than in American and European countries [1, 3–5], and prevalence rates of somatoform disorders are inconsistent [6, 7]. For example, the 12-month prevalence of anxiety disorders in China was 5.0% compared to 15.3% in Germany [8, 9] and for major depressive episodes was 3.6% in China and 6% in Germany [8, 10]. Cultural, linguistic and methodological aspects could contribute to explaining the differences in prevalence rates of disorders. According to a literature review [4], the lower prevalence of major depressive disorders that persisted in East/Southeast Asia compared to other regions of the world still remained, even after adjusting for methodological differences. The study showed evidence that cross-national differences may reflect either true prevalence differences or the cross-cultural insensitivity of diagnostic criteria such as the *Diagnostic and Statistical Manual of Mental Disorders* [11] (DSM) and the *International Classification of Diseases* [12] (ICD) [13]. A deeper cross-cultural examination of these differences is overdue.

The Patient Health Questionnaire [14], which includes the somatic symptom severity scale (PHQ-15), the depressive symptom severity scale (PHQ-9) and the seven-item Generalized Anxiety Disorder (GAD-7) scale, screens, identifies and measures the severity of most common mental disorders and is one of the most commonly used instruments to assess psychological disorders. It is a short self-report questionnaire based on the diagnostic criteria of the DSM-IV and its scales also have a high level of suitability for the newly developed DSM-V [11], with the American Psychiatric Association (APA) recommending them for measuring the severity of depression, anxiety disorders and somatoform disorders [15]. In both Western and Chinese general populations it showed good reliability and validity of scores [16–22]. Furthermore, taking into consideration that Western psychologization is stronger than Chinese somatization, this self-completed questionnaire had the advantage of revealing more psychological distress than in interviews with the Chinese population [23].

In order to accurately compare the cross-cultural prevalence of these mental disorders, diagnostic measurements such as the PHQ must be measurement invariant across cultures and languages (comparable both cross-culturally and cross-linguistically). DSM- or ICD-based diagnostic measures were criticized as not being culturally sensitive enough due to culture-specific forms of disorders [24]. Cultural differences in scale scores can also result from differences in the understanding of certain concepts, translation problems, frequency of word use or other measurement errors, and the potentially biased items should be identified before comparison [25]. Despite the widespread use of the PHQ, cross-cultural examination of its measurement invariance has been mostly neglected and such examinations have scarcely been made between mainland Chinese and Western samples.

The commonly used measurement equivalence approach (also called measurement invariance) is confirmatory factory analysis (CFA) and this offers a robust statistical framework for testing measurement invariance. The most frequently assessed levels of measurement invariance included configural, metric and scalar invariance, which refer to different model parameters of a measurement model and build on each other in a hierarchical structure. Configural invariance, the least restrictive form of measurement invariance, is present when the number of factors and the pattern of the factor loadings between the latent variables and indicators in the compared groups are similar. When the factor loadings of items are also invariant across groups, then metric invariance could be assumed. Scalar invariance exists if, additionally, the intercepts of the indicator variables are identical [26]. Scalar invariance or at least partial measurement invariance, which is declared when at least two items per latent variable (i.e., factor loadings, factor intercepts) are found to be invariant, is a prerequisite for the comparison of latent mean values obtained from subsamples [27–29].

In previous studies, measurement invariance of the PHQ-15 with a bifactor model (one general somatic symptom factor and four orthogonal-specific symptom factors of pain, gastroenterology, cardiovascular and fatigue symptoms) could be confirmed with samples of college students from Germany and Switzerland [30], between German and migrants [31] and between patient samples from Germany and the Netherlands, but not between Chinese patient samples and Western (German and Dutch) patient samples [32]. Measurement invariance with a one-factor model was confirmed between primary care patients of native-born Germans, Russian-speaking immigrants and native-born Russians [33]. So far, hardly any studies have explored measurement invariance of the PHQ-15 in samples from mainland China and Western countries.

Previous studies have predominantly been able to confirm a one-factor structure of the PHQ-9 with different samples across cultures or migrants in Western countries and found measurement invariance of the scale in six ethnic groups in the Netherlands, in diverse college populations in the USA and in first- and second-generation migrants of the German population [34–38]. However, the items “sleep problems”, “appetite changes” and “anhedonia” showed cross-cultural measurement biases when comparing Turkish immigrants and Germans, and the item “psychomotor problems” seemed to be culturally biased in Surinam Dutch males compared to Dutch males. A bifactor structure (“somatic factor” and “non-somatic factor”) could be confirmed in a German study with samples across gender [39] and in a Japanese study between clinical and nonclinical samples [40].

Existing evidence demonstrates that the GAD-7 has good psychometric properties and shows reliability and validity of scores as a measure of anxiety in the German general population [19] and in various samples in Chinese primary care [21, 41]. So far, only a few studies have examined cross-cultural measurement invariance of the GAD-7. A study by Parkerson, Thibodeau, Brandt, Zvolensky, and Asmundson [42] has confirmed a revised unitary model of the GAD-7 and found that Black/African Americans with high GAD symptoms scored lower on the GAD-7 than White/Caucasian Hispanic participants. It indicated that the GAD-7 is not culturally sensitive enough and the lower prevalence rate for the Black/African American sample could reflect cross-cultural measurement biases related to the diagnostic instrument rather than true differences in GAD symptoms. It is still unclear whether such cross-cultural measurement biases also exist in the epidemiological comparison of cultural groups from China and Western countries.

To address the above-mentioned limitations of current studies in examining cross-cultural measurement biases of the PHQ in samples from mainland China and Western countries, we conducted this study to investigate measurement invariance of the PHQ-15, PHQ-9 and GAD-7 across Chinese and Western (represented by Germany) cultures. We investigated student samples because of the advantage of comparability in educational status, age and other psychosocial aspects, but also the different cultural backgrounds. Based on previous research, we expected there to be measurement invariance of the PHQ-9 between the two cultures but predicting the same for the PHQ-15 may be problematic. Due to lack of previous studies, we did not make a hypothesis about the intercultural measurement invariance of the GAD-7. Differences in latent means for somatic symptoms, depression and anxiety syndromes were also assessed if scalar measurement invariance across cultures was demonstrated. Investigating the cross-cultural equivalence of the PHQ-15, PHQ-9 and GAD-7 has high relevance to the diagnosis of mental disorders and is a prerequisite for cross-cultural comparisons.

## Methods

### Participants

The online data used in the present study are from a dataset collected in a project for intercultural comparison of willingness to seek psychological help [43]. The data were collected in Germany and China in August 2016 and the collection lasted for 6 months. German students at the University of Marburg (total number of students: 26,355) were invited to take part in the survey via the university email list. To increase the interest to participate, they could be entered into a draw for vouchers worth 20 euros. Chinese students at the University of Zhengzhou in China were recruited on “WeChat”, a popular social media platform used by most Chinese students, and they received no financial reward. Chinese students who were in the WeChat groups of various affiliated faculties (e.g., Economics and Electronic Information Engineering: 9156 students) were invited to participate in this study. After the application of exclusion criteria (no migration background; a minimum scale processing time of 10 min), the Chinese sample available for analysis decreased from 566 to 413 and the German sample from 456 to 416. The demographic characteristics of the two groups are summarized in Table 1.^1^ The study was approved by the ethics committee of the Faculty of Psychology at the University of Marburg (approval number: 2016-19 k).

**Table 1:** Participant Demographic Characteristics

Variables	German Students (*n* = 416)	Chinese Students (*n* = 413)	*p*-value	Effect size
Sex [female] (%)	294 (70.7%)	250 (60.5%)	.001	*φ* = .15
Age (Mean ± SD; range)	23.85 ± 4.66; 18–58	20.79 ± 2.57; 17–39	*p* < .001	Cohen’s *d =* .81
Current Academic Degree			p < .001	Cramer’s V = .78
Bachelor (%)	183 (44%)	391 (94.7%)		
Master (%)	83 (20%)	7 (1.7%)		
Ph.D. (%)	17 (4.1%)	4 (1.0%)		
Others (%)	137 (32%)	11 (2.7%)		

*Note.* “Other” refers to a combined Bachelor’s and Master’s degree program. The comparisons of sex and academic degree were calculated using

Chi-squared test. The age comparison was calculated using T-test

### Assessment instruments

#### PHQ-15

The PHQ-15 was used to assess and diagnose somatoform disorders [44] and includes 15 prevalent somatic symptoms that represent the most common symptoms observed in primary care that typically cannot be fully explained by a diagnosed general medical condition. Two of the items were from the depression subscale of the PHQ-9 (“*Trouble falling or staying asleep, or sleeping too much*”; “*Feeling tired or having little energy*”). Three response categories were offered: “not bothered at all”, “bothered a little” or “bothered a lot”. The total score ranged from 0 to 30. The reliability and validity of the scores were supported by studies both in German and Chinese populations [17, 18, 22].

#### PHQ-9

The PHQ-9 was used to assess and diagnose depression [45]. The participants responded on a four-point Likert scale and the total score ranged from 0 to 27. The PHQ-9 has good psychometric properties and includes high sensitivity for depressive disorders and good specificity for screening of patients with depression in both Chinese and German general populations [20, 46] and in their corresponding primary care populations [16, 17]. The PHQ-9 was considered superior to other self-rating instruments for the detection of depressive disorders [17].

#### GAD-7

The seven-item GAD-7 was developed to identify potential patients with a generalized anxiety disorder [47] and to assess the severity of symptoms of general anxiety because of its good operating characteristics for anxiety disorders [48]. Participants indicated agreement with the presence of symptoms such as “*Feeling nervous, anxious or on edge*” and “*Not being able to stop or control worrying*” on a four-point Likert scale ranging from 0 (*not at all*) to 3 (*nearly every day*). The total score ranges from 0 to 21.

### Translation

The German validated versions of the PHQ-15, PHQ-9 and GAD-7 [14] were used in the German sample and the translation was done according to “state of the art criteria” using the translation/retranslation method. The Chinese versions of the PHQ-15 [22], PHQ-9 [46] and GAD-7 [49] were also validated in previous studies and the translation followed the customary translation/back-translation method.

### Statistical analysis

First, SPSS (version 25, IBM, Armonk, USA) software was used for checking the descriptive statistics (means, standard deviations, skewness and kurtosis of the sum scores and evidence of internal consistencies for each scale and each sample), and then we used the software program M*plus* v7.4 [50] for further data analysis. We examined separate measurement models for each group using confirmatory factor analysis (CFA). To assess the model fit we used χ^2^ difference tests, as recommended by Hu and Bentler [51]. Because the χ^2^ difference test is sensitive to sample size, other common indices to assess the goodness of model fit were also used: comparative fit index (CFI), root mean square error of approximation (RMSEA), standardized root mean residual (SRMS) and difference in CFI between the base model and the constrained model (ΔCFI). The following cutoff values were used: CFI ≥ .90 [52], RMSEA ≤ .08 and SRMR ≤ .08 [53].

Then the step-up approach was applied to add a series of increasingly stringent equality constraints to the models [27, 54]. The configural invariance of the baseline model was estimated as the starting point of the multiple group comparisons, in which all parameters (factor loadings and intercepts of indicators) vary freely. We investigated whether the construct was similarly displayed in different groups, meaning that both the number of specified factors and the indicators that load on the factors should be comparable. In the next step, the metric invariance was checked by constraining the factor loadings of indicators to be equal. Then the scalar invariance, the next highest form of measurement invariance, was assessed by additionally constraining intercepts of indicators to be equal. After gradual equality constraining of the parameters across the groups, these models were compared with the baseline model. The decision on whether a model was accepted or not was made according to the χ^2^ difference test [51]. As the χ^2^ value was dependent on sample size, Cheung and Rensvold [55] suggested that the difference in CFI between the baseline model and the constrained model should not be more than 0.01. If the full measurement invariance cannot be confirmed, partial invariance should be examined [28]. The constrained model based on the modification indices was subsequently examined by releasing the equality constraints in descending order for misspecified items. At least two loadings or intercepts should be equal between groups in order to establish partial measurement invariance. If evidence for scalar invariance or at least partial scalar invariance^2^ exists, then the latent means of samples could be compared [27, 28].

## Results

### Descriptive statistics

Means, standard deviations, skewness and kurtosis of the sum scores and evidence of internal consistencies for each scale and each sample are presented in Table 2. According to the cutoff values (skewness ≤3, kurtosis ≤8) recommended by Kline (2010), skewness and kurtosis indicated a normal distribution in the samples. The internal consistency of the scores was at least good (> .70). Items 2 (“back pain”) and 9 (“fainting spells”) in the German version of the PHQ-15 showed a small correlation (< .10) with other items of the scale, mainly because of very low or very high base rates compared to other symptoms. Despite these findings, we first tested the original models using CFA.

**Table 2:** Means, Standard Deviations, Skewness, Kurtosis, and Internal Consistency across Scales and Samples

	German Students						Chinese Students				
Scale	*M*	*SD*	Skew	Kurt	α		*M*	*SD*	Skew	Kurt	α
PHQ-15	7.39	4.49	.77	.44	.76		6.87	4.67	.75	.22	.83
PHQ-9	6.77	4.84	.76	.09	.85		6.99	4.76	1.01	1.49	.88
GAD-7	6.23	4.27	.80	.06	.87		5.38	4.00	1.18	1.93	.90

*Notes*. PHQ-15 = Patient Health Questionnaire-15 Physical Symptoms; PHQ-9 = Patient Health Questionnaire-9 Depression Symptoms; GAD-7 = Generalized Anxiety

Disorder 7-item Scale; Skew = Skewness; Kurt = Kurtosis

### Measurement invariance of the PHQ-15

#### Single-group CFA

Results from CFA are presented in Table 3. The unidimensional model of the PHQ-15 was examined first, which assumes only one latent factor (model A). In both groups, the PHQ-15 resulted in acceptable SRMR but poor CFI and RMSEA values (Chinese group: CFI = .827, RMSEA = .079, 90% CI [.070, .089], SRMR = .057; German group: CFI = .716, RMSEA = .086, 90% CI [.077, .096], SRMR = .065). This means that a one-factor solution does not fit the samples of Chinese and German students. Then we tried the hierarchical measurement model with four first-order latent factors and a second-order latent factor (model B) recommended by Mewes et al. [31], which was based on the criteria for somatoform disorders and physical complaints of depressive disorders in ICD-10 and DSM-IV. The four factors are as follows: pain symptoms (item 2 “back pain,” item 3 “pain in your arms, legs or joints,” item 4 “menstrual cramps or other problems with your periods”, item 5 “pain or problems during sexual intercourse”, item 6 “headaches”), gastrointestinal symptoms (item 1 “stomach pain”, item 12 “constipation, loose bowels or diarrhea”, item 13 “nausea, gas or indigestion”), cardiovascular symptoms (item 7 “chest pain”, item 8 “dizziness”, item 9 “fainting spells”, item 10 “feeling your heart pound or race”, item 11 “shortness of breath”) and fatigue symptoms (item 14 “trouble sleeping”, item 15 “feeling tired or having low energy”) (see Supplementary Material, Table S1). The model with four first-order latent factors and a second-order latent factor achieved an acceptable fit for both samples in terms of RMSEA (Chinese group: CFI = .936, RMSEA = .050, 90% CI [.039, .061], SRMR = .042; German group: CFI = .914, RMSEA = .049, 90% CI [.038, .060], SRMR = .049). Baseline models for analysis of measurement invariance between cultures could be established.

**Table 3:** Fit Indices from Comparative Factor Analysis (CFA) and Invariance Analyses between Groups for the PHQ-15

	χ2 *(df)*	CFI	RMSEA [90% CI]	SRMR	ΔCFI	Δχ2 *(df)*
model A. one-factor model						
German Students	369.242 (90)	.716	.086 [.077, .096]	.065		
Chinese Students	323.274 (90)	.827	.079 [.070, .089]	.057		
model B. four-factor model						
German Students	168.941 (84)	.914	.049 [.038, .060]	.049	.016	200.301 (6)
Chinese Students	170.681 (84)	.936	.050 [.039, .061]	.042	.015	152.593 (6)
Multiple group CFA model B						
Configural invariance	339.622 (168)	.926	.050 [.042, .057]	.045		
Metric invariance	410.344 (183)	.902	.055 [.048, .062]	.071	.024	70.722 (15)
λ_9_ free	393.042 (182)	.909	.053 [.046, .060]	.063	.023	53.420 (14)
λ_9,_ λ_10_ free	383.574 (181)	.913	.052 [.045, .059]	.059	.013	43.952 (13)
λ_9,_ λ_10,_ λ_11_ free	371.885 (180)	.918	.051 [.043, .058]	.056	.008	32.263 (12), *p >* .001
Scalar invariance	556.111 (195)	.845	.067 [.060, .073]	.067	.073	184.226 (15)
τ_10_ free	501.412 (194)	.868	.062 [.059, .069]	.062	.050	129.527 (14)
τ_10,_ τ_2_ free	482.435 (193)	.876	.060 [.053, .067]	.060	.042	110.55 (13)
τ_10,_ τ_2,_ τ_5_ free	469.157 (192)	.881	.059 [.052, .066]	.059	.037	97.272 (12)
τ_10,_ τ_2,_ τ_5,_ τ_12_ free	451.368 (191)	.887	.058 [.051, .065]	.058	.031	79.483 (11)
τ_10,_ τ_2,_ τ_5,_ τ_12,_ τ_9_ free	440.687 (190)	.892	.056 [.050, .063]	.056	.026	68.802 (10)
τ_10_, τ_2_, τ_5,_ τ_12,_ τ_9,_ τ_6_ free	430.746 (189)	.896	.056 [.049, .062]	.054	.022	58.864 (9), *p <* .001

*Notes*. PHQ-15 = Patient Health Questionnaire-15 Physical Symptoms; Item 2 = ‘back pain’; item 5 = ‘pain or problems during sexual intercourse’; item 6 = ‘headaches’; item 9 = ‘fainting spells’; item 10 = ‘feeling your heart pound or race’; item 11 = ‘shortness of breath’; item 12 = ‘constipation, loose bowels, or diarrhea.’ All χ^2^ tests and Δχ2 were significant, *p* < .001

#### Measurement invariance between cultures

After confirming the superiority of model B compared to model A, measurement invariance analysis between cultures was performed. The testing results of measurement invariance for the PHQ-15 are presented in Table 3. The baseline model of the PHQ-15 showed acceptable configural invariance (CFI = .926, RMSEA = .050, 90% CI [.042, .057], SRMR = .045). In the next step, the metric invariance was tested by constraining the factor loadings to be equal. The fit of the metric invariance was poor, with a decrease in CFI of more than 0.01 (CFI = .902, RMSEA = .055, 90% CI [.048, .062], SRMR = .071, ΔCFI = .024). Modification indices indicated that the loading of items 9 (fainting spells), 10 (feeling your heart pound or race) and 11 (shortness of breath) differed across the groups. After releasing the constraints for these items in descending order, the fit of this modified model was acceptable (CFI = .918, RMSEA = .051, 90% CI [.043, .058], SRMR = .056, ΔCFI = .008). Then the factor intercepts were constrained to be equal and the scalar invariance was shown to be poor, with a CFI of .845 and a drop in CFI of more than 0.01 (ΔCFI = .073). The modification indices showed that the intercepts of items between the two groups were invariant. After releasing the equality constraints for items 10 (feeling your heart pound or race), 2 (back pain), 5 (pain or problems during sexual intercourse), 12 (trouble sleeping), 9 (fainting spells) and 6 (headaches) in descending order, the fit of this modified model for checking partial scalar invariance was still unacceptable, with a poor model fit and a drop in CFI of more than 0.01 (CFI = .897, ΔCFI = .021). Hence, the partial scalar invariance of the four-factor model between the groups could not be established and comparison of the latent means could not be conducted.

### Measurement invariance of the PHQ-9

#### Single-group CFA

Similar to the PHQ-15, we first examined the fit of the one-factor model of the PHQ-9 using CFA in the two groups (Table 4). The one-factor model of the PHQ-9 had acceptable CFI and SRMR in both groups but poor RMSEA, with values of more than .08 (Chinese group: CFI = .951, RMSEA = .082, 90% CI [.065, .099], SRMR = .038; German group: CFI = .900, RMSEA = .104, 90% CI [.088, .120], SRMR = .051). Therefore we tried a two-factor-solution, which was suggested by Petersen et al. [39]. The two factors included “somatic” (e.g., sleep disturbances, fatigue and appetite changes) and “non-somatic” items (e.g., depressed mood, lack of interest and suicidal ideation). The model with two latent factors afforded a good fit in both samples (Chinese group: CFI = .957, RMSEA = .078, 90% CI [.061, .096], SRMR = .037; German group: CFI = .969, RMSEA = .059, 90% CI [.040, .077], SRMR = .033). A baseline model for analysis of measurement invariance between the two groups could be established.

**Table 4:** Fit Indices from Comparative Factor Analysis (CFA) and Invariance Analyses between Groups for the PHQ-9

	χ2 *(df)*	CFI	RMSEA [90% CI]	SRMR	ΔCFI	Δχ2 *(df)*
model A. one-factor model						
German Students	147.851 (27)	.900	.104 [.088, .120]	.051		
Chinese Students	101.362 (27)	.951	.082 [.065, .099]	.038		
model B. two-factor model						
German Students	63.070 (26)	969	.059 [.040, .077]	.033		84.781
Chinese Students	91.453 (26)	.957	.078 [.061, .096]	.037		9.909, p *>* .001
Multiple groups CFA models						
Configural invariance	154.523 (52)	.962	.069 [.057, .082]	.035		
Metric invariance	229.833 (61)	.938	.082 [.071, .093]	.082	.024	75.310 (9)
λ_8_ free	185.565 (60)	.954	.071 [.060, .083]	.064	.008	31.042 (8)
λ_8,_ λ_1_ free	174.288 (59)	.958	.069 [.057, .081]	.068	.004	19.765 (7)
λ_8,_ λ_1,_ λ_3_ free	169.246 (58)	.959	.068 [.056, .080]	.061	.002	14.723 (6), p *>* .001
Scale invariance	302.171 (65)	.913	.094 [.083, .105]	.085	.046	132.925 (7)
τ_8_ free	216.478 (64)	.944	.076 [.065, .087]	.063	.015	47.232(6)
τ_8_, τ_4_ free	191.586 (63)	.953	.070 [.059, .082]	.061	.006	22.340 (5)
τ_8_, τ_4_, τ_1_ free	179.934 (62)	.957	.068 [.056, .079]	.061	.002	10.688 (4), p *>* .001

*Note.* PHQ-9 = Patient Health Questionnaire-9 Depression Symptoms; Item 1 = ‘lack of interest’; item 3 = ‘sleep difficulties’; item 4 = ‘feeling tired or having little energy’; item 8 = ‘moving or speaking slowly, or fretful.’ All χ2 tests and Δχ2 were significant, p < .001

#### Measurement invariance between cultures

We examine the measurement invariance across cultures with model B because of its better model fit than model A. The model specifications for the PHQ-9 are displayed in Table 4. The global fit for the configural model was good (CFI = .962, RMSEA = .069, 90% CI [.057, .082], SRMR = .035). Then, item loadings were constrained to be equal in the metric invariance model. The global fit was poor, with RMSEA and SRMR bigger than .080 and ΔCFI bigger than .01. Modification indicated that loadings of items 8, 1 and 3 were invariant. The loading of items 1 (lack of interest) and 8 (moving or speaking slowly, or fretful) was higher in the Chinese sample and for item 3 (sleep difficulties) was higher in the German sample. Partial metric invariance was established by allowing the loadings of these items to vary in descending order (CFI = .959, RMSEA = .068, 90% CI [.056, .080], SRMR = .061, ΔCFI = .002). At the level of scalar invariance, RMSEA and SRMR were also bigger than .08 and the drop in CFI was larger than .01. After releasing the equality constraints of items 8, 4, and 1 in descending order, partial scalar invariance could be established (CFI = .957, RMSEA = .068, 90% CI [.056, .079], SRMR = .061, ΔCFI = .002).

#### Latent mean comparison

Comparison of the latent means was based on five invariant items (items 2, 5, 6, 7 and 9) and the German sample was used as the reference group. The Chinese students had a higher latent mean than German students, which means that Chinese students have more depressive symptoms than German students, but the mean difference was not significant (*z* = .344, *d* = .153, *p* = .365).

### Measurement invariance of the GAD-7

#### Single-group CFA

CFA of the original one-factor model demonstrated an acceptable global fit in the sample of Chinese students, but the RMSEA indicated a poor fit in the sample of German students (Table 5). Modification indices suggested that the error terms of items 5 (“being so restless that it is hard to sit still”) and 6 (“becoming easily annoyed or irritated”) were correlated in both samples, which was similar to the findings from Parkerson et al. (2015). To improve the comparability of the two groups, correlation between the two item errors was allowed and this produced an acceptable RMSEA for the sample of German students. At the same time, the global model fit for the sample of Chinese students was also improved significantly (Δχ^2^ (df) = 15.219 (1), *p* < .001*)*.

**Table 5:** Fit Indices from Comparative Factor Analysis (CFA) and Invariance Analyses between Groups for the GAD-7

	χ2 *(df)*	CFI	RMSEA [90% CI]	SRMR	ΔCFI	Δχ2 *(df)*
Single group CFA − original one-factor model						
German Students	53.671 (14)	.967	.083 [.060, .106]	.035		
Chinese Students	49.414 (14)	.977	.078 [.055, .102]	.028		
Single group CFA (θ_5, 6_ free)						
German Students	44.662 (13)	.974	.077 [.053, .102]	.031	.013	9.009, *p >* .001
Chinese Students	34.195 (13)	.986	.063 [.038, .089]	.023	.009	15.219
Multiple group CFA models (θ_5, 6_ free)						
Configural invariance	87.866 (27)	.978	.074 [.057, .091]	.030		
Metric invariance	127.041 (34)	.966	.081 [.066, .097]	.073	.012	39.175 (7)
λ_1_ free	107.649 (33)	.973	.074 [.059, .090]	.048	.005	19.783 (6), p *>* .001
Scale invariance	185.052 (39)	.947	.095 [.082, .109]	.063	.026	77.403 (6)
τ_4_ free	164.669 (38)	.954	.090 [.076, .104]	.058	.019	57.020 (5)
τ_4_, τ_1_ free	137.262 (37)	.963	.081 [.067, .096]	.052	.010	29.613 (4)
τ_4_, τ_1,_ τ_2_ free	120.930 (36)	.969	.075 [.061, .090]	.052	.004	13.281 (3), p *>* .001

*Note.* GAD-7 = Generalized Anxiety Disorder 7-item; Item 1 = ‘feeling nervous, anxious, or on edge’; item 2 = ‘not being able to stop or control worrying’; item 4 = ‘trouble relaxing.’ All χ2 tests and Δχ2 were significant, *p* < .001

#### Measurement invariance between cultures

The results of tests of the measurement invariance of the GAD-7 are presented in Table 5. The baseline model of the GAD-7 demonstrated a good global fit (CFI = .978, RMSEA = .074, 90% CI [.057, .091], SRMR = .030) and its configural invariance was confirmed. At the level of metric invariance, the RMSEA was larger than .08 and the drop in CFI was larger than .01 (RMSEA = .081, 90% CI [.066, .097], ΔCFI = .012). Modification indices indicated that the loading of item 1 was not invariant. The loading of item 1 was higher in the German sample than in the Chinese sample. A modified model by releasing the equality constraints for item 1 provided a good fit and the assumption of metric invariance held (CFI = .973, RMSEA = .074, 90% CI [.059, .090], SRMR = .048, ΔCFI = .005). On testing for scalar invariance, the RMSEA was larger than .08 and the drop in CFI was larger than .01 (RMSEA = .095, 90% CI [.082, .109], ΔCFI = .026). Modification indices showed that the intercepts of items 4, 1 and 2 were higher in the German sample than the Chinese sample. By releasing the equality constraints of items 4, 1 and 2 in descending order, the global fit of this model was improved (CFI = .969, RMSEA = .075, 90% CI [.061, .090], SRMR = .052, ΔCFI = .004) and partial scalar invariance was established.

#### Latent mean comparison

Comparison of the latent means was based on four invariant items (items 3, 5, 6 and 7) and the sample of German students was used as the reference group. The Chinese students had a lower latent mean than German students on the GAD-7, but the difference was not significant (*z* = −.023, *d* = .023, *p* = .759).

## Discussion

In our study, we examined the cross-cultural measurement invariance of the PHQ-15, PHQ-9 and GAD-7 by comparing two cultural groups of students, one from mainland China and the other from Germany. The results demonstrated that the original one-factor model of the PHQ-15 fitted neither of the groups. The bifactor model (one general factor and four orthogonal symptom-specific factors) of the PHQ-15 showed a better model fit in both groups but only configural and metric invariance between the groups could be confirmed, therefore it is not recommended for the cross-cultural comparison of means. The PHQ-9 and GAD-7 had the same factor structure in the two groups and showed partial scalar invariance. This means that although these scales show differences on individual items, they are generally comparable across the two cultural groups of students, which provides the possibility for cross-cultural comparative studies in the future.

We could not confirm the bifactor model (one general factor and four orthogonal symptom-specific factors) of the PHQ-15 with the cross-cultural student samples as suggested by Mewes et al. [31]. We also did not find full metric and partial scalar invariance. The possible reason for this could be that the samples included in our study have a greater difference in cultural background. Our result corresponded with the findings of an earlier cross-cultural study [32], which also could not confirm measurement invariance of the PHQ-15 between Chinese and German samples of outpatients. In our study, the pattern of variant items at the level of metric and scalar invariance across groups was mixed. Chinese students are more likely to endorse items 10 (“feeling your heart pound or race”), 11 (“shortness of breath”), 9 (“fainting spells”) and 12 (“constipation, loose bowels, or diarrhea”) and German students are more likely to endorse items 5 (“pain or problems during sexual intercourse”), 6 (“headaches”) and 2 (“back pain”). Regarding the differences between individual items, there was a slight attempt in previous studies to focus on the influence of culture on shaping somatic awareness. A possible explanation for the differences could be that the levels of interoceptive accuracy and somatic awareness between people from Western and non-Western countries are different [56], and this phenomenon could be more strongly expressed on certain somatic symptoms in cross-cultural comparisons. Somatic awareness is a top-down process that is driven by attention, beliefs and expectations [57, 58] and these factors may affect the evaluation of the importance of different physical symptoms in different cultures. Linguistics is an important approach for studying this cultural difference. For example, future research could focus on whether certain body parts are used more than others in the description of physical states in the Chinese and German languages. In terms of methodology for testing a series of equality constraints on parameters in measurement models such as the PHQ-15 that have a complex structure across groups, multi-group CFA has the limitation that “the standard model fit criteria do not represent ‘golden rules’“ [59]. An alternative approach could be the multi-group exploratory structural equation modeling recommended by Marsh et al. [60], which can test measurement invariance directly and is viable for scales with a complex structure.

Consistent with the results of previous studies by Doi et al. [61] and Petersen et al. [39], a bifactor structure of the PHQ-9 could be confirmed in our study. We found partial metric and partial scalar invariance of the PHQ-9 across the two cultural groups. Chinese students are more likely to endorse items 1 (lack of interest) and 8 (moving or speaking slowly, or being fidgety) and German students are more likely to endorse items 3 (sleep difficulties) and 4 (fatigue). The higher score on item 1 (lack of interest) is consistent with the results of the study by Leung [62], which found that East Asian students who share the Confucian culture (high regard for academic achievement) displayed relatively negative attitudes toward learning even though they outperformed Western students. Hau and Ho [63] have reviewed the previous studies and found that Chinese students are more likely to study under external pressure and have lower interest in studying. Regarding “sleep problems”, our study could support Parker, Cheah and Roy’s [64] finding that insomnia is not being overrepresented in the Chinese sample, although many Asian psychiatrists have seen it as one of the most common reasons for depressed Chinese to seek help. It appears to be a true concomitant of depression and not distinctly culturally determined.

Chinese students may have a higher prevalence of depression than other populations in China because they are more open and inclined to express emotional distress. This is in line with the comparison of latent means of the two groups, showing that German students did not express more depressive symptoms than Chinese students, although previous studies have found that the prevalence of depression disorders was lower in Southeast Asia (including China) than in Western Europe [4, 65]. To use the PHQ-9 in the general Chinese population, who are not necessarily willing to report the emotional symptoms of depression or are less aware of them, a lower cut-off value would be advisable in order to maximize the detection of people with depression [66].

Partial scalar invariance of the original one-factor model of the GAD-7 could be confirmed across groups with Chinese and German students. The difference across groups indicated that German students are more likely to report anxiety symptoms such as “feeling anxious” (item 1), “not being able to stop worrying” (item 2) and “trouble relaxing” (item 4). But these differences were not significant and the latent means of the two groups did not differ, which means that German students did not have significantly higher levels of general anxiety than Chinese students. This is not consistent with the results of previous studies, which show that non-Western cultures have less risk of anxiety disorder [3, 67]. In Asian countries, culture-specific anxiety symptoms such as shame [68] were not included in the GAD-7 and it is unclear whether such aspects play a role in the measurement of general anxiety severity because empirical research is lacking.

### Limitations

This study has some limitations that should be considered. First, our study was conducted in samples of college students, which controlled for other non-cultural factors contributing to the results (e.g., education), but it is unclear whether the findings of this study can be generalized to other population groups. It could be more difficult to establish measurement invariance in other populations across cultures because the younger generation who grew up after China adopted policies of reform and greater openness were more influenced by Western lifestyle and values and may have a different pattern of expressing emotional distress than the older generation in China. Second, we used online recruitment of the sample, which has the advantage of being economical and fast but also the disadvantage of the self-selection effect of participants. For organizational reasons, the Chinese students did not receive financial compensation for participating in the study and this could lead to bias in the data. Furthermore, the scales were found to be partially measurement invariant and to fulfill the prerequisite for comparison of latent means by including only unbiased items, which can lead to shortcomings in the interpretation of cross-cultural comparisons.

## Conclusions

In summary, our findings imply that the PHQ-9 and GAD-7 could be considered as construct invariant for students across Chinese and German cultures, with individual items showing cultural differences, and thus could be used for cross-cultural comparison. The PHQ-15 did not show scalar invariance. Full scalar invariance is generally difficult to find, especially across strongly contrasting cultures. This may be due to translation problems for certain items, cultural bias in understanding certain concepts and problems with the method for testing measurement invariance. Intercultural cooperation should be encouraged in order to improve the diagnostic instruments, which are more sensitive to culturally specific symptoms. Future studies may consider alternative approaches to test measurement invariance and more research into the influence of culture on shaping somatic awareness is required. Furthermore, it is necessary to examine the universality of the scales across diverse aged populations. Previous studies demonstrated that there are qualitative differences in the symptom presentation of depression and anxiety in younger and older adults, and that the different presentations of depression and anxiety in older adults are not fully assessed by the current measures of depression and anxiety [69, 70]. Our study is one of the first to investigate the measurement invariance of the frequently used PHQ-15, PHQ-9 and GAD-7 in large groups in China and Germany, which suggests that the constructs of a subject (e.g., somatic symptoms) could vary in its expression in different cultural contexts and that measurement equivalence of the measurement instrument should be ensured in comparative cultural studies.

## Supplementary information

**Additional file 1: Table S1**. Items of the four first-order latent factors of the PHQ-15

## Data Availability

The datasets used and/or analysed during the current study are available from the corresponding author on reasonable request.

## Abbreviations

PHQ-15: Patient Health Questionnaire-15 Physical Symptoms
PHQ-9: Patient Health Questionnaire-9 Depression Symptoms
GAD-7: Generalized Anxiety Disorder 7-item scale
CFI: Comparative fit index
RMSEA: Root mean square error of approximation
SRMS: Standardized root mean residual
ΔCFI: The difference in CFI between the base model and the constrained model

## Footnotes

1 “Since the demographic data of the two samples was significantly different, propensity score matching (by gender and age) was performed to exclude the test effect of gender and age for the purpose of data analysis. However, the results did not differ from those obtained prior to performing the propensity score matching test. The results of the comparison between the underlying structure and the invariant items of the scales remained unchanged. Therefore, we presented the results without conducting propensity score matching, owing to the large sample size” [43].

2 Partial measurement invariance is declared when at least two items per latent variable (i.e., factor loadings, factor intercepts) are found to be invariant [29].
