# Supplementary material for: Are comparisons of mental disorders between Chinese and German students possible? An examination of measurement invariance for the PHQ-15, PHQ-9 and GAD-7
Source: BMC Psychiatry. 2020 Oct 1;20:480. doi: 10.1186/s12888-020-02859-8 (PMC7531122; doi:10.1186/s12888-020-02859-8)
Supplement: Supplementary file 1 — Additional file 1: Table S1. Items of the four first-order latent factors of the PHQ-15 [file 12888_2020_2859_MOESM1_ESM.docx]

***Additional file 1***

Additional file Table S1. Items of the four first-order latent factors of the PHQ-15

| Item | Factor 1. Pain symptoms |
| --- | --- |
| 2 | Back pain |
| 3 | Pain in your arms, legs, or joints (knees, hips, etc.) |
| 4 | Menstrual cramps or other problems with your periods |
| 5 | Pain or problems during sexual intercourse |
| 6 | Headaches |
|  | Factor 2. Gastrointestinal symptoms |
| 1 | Stomach pain |
| 12 | Constipation, loose bowels, or diarrhea |
| 13 | Nausea, gas, or indigestion |
|  | Factor 3. Cardiovascular symptoms |
| 7 | Chest pain |
| 8 | Dizziness |
| 9 | Fainting spells |
| 10 | Feeling your heart pound or race |
| 11 | Shortness of breath |
|  | Factor 4. Fatigue symptoms |
| 14 | Trouble sleeping |
| 15 | Feeling tired or having low energy |
